# Vitamin C improves 28-day survival in patients with sepsis-associated acute kidney injury in the intensive care unit: a retrospective study

**DOI:** 10.3389/fnut.2025.1600224

**Published:** 2025-06-05

**Authors:** Yang He, Jinglan Liu

**Affiliations:** ^1^Yichang Central Department of Critical Care Medicine, The First College of Clinical Medical Science, China Three Gorges University, Yichang Central People’s Hospital, Yichang, Hubei, China; ^2^Yichang Central People’s Hospital, Yichang, Hubei, China

**Keywords:** vitamin C supplementation, sepsis-associated acute kidney injury (SA-AKI), 28-day mortality, MIMIC-IV database, ICU

## Abstract

**Background:**

Vitamin C, a water-soluble essential micronutrient, exhibits multifaceted physiological roles including immune modulation and enhanced resistance to infectious pathogens. Evidence suggests that hypovitaminosis C is associated with adverse clinical outcomes in critically ill populations, with notably high prevalence observed in acute kidney injury patients. This retrospective study aimed to evaluate the potential association between vitamin C supplementation during intensive care unit admission and improved clinical outcomes, specifically in sepsis-associated acute kidney injury (SA-AKI).

**Methods:**

Utilizing data from the Medical Information Mart for Intensive Care IV (MIMIC-IV), a repository of ICU patient records from Beth Israel Deaconess Medical Center (United States), we identified patients diagnosed with SA-AKI. Participants were stratified into two cohorts: those receiving intravenous vitamin C supplementation during ICU stay (vitamin C group) and those without supplementation (non-vitamin C group). Primary outcomes, including in-hospital mortality, were evaluated using Kaplan–Meier survival curves, Cox proportional hazards regression models, and subgroup analyses. Propensity score matching (PSM) was employed to mitigate potential confounding. Secondary outcomes encompassed 28-day mortality.

**Results:**

Among 16,140 patients diagnosed with SA-AKI, 589 received vitamin C supplementation, while 15,551 did not. Kaplan–Meier analysis revealed a significant divergence in survival probabilities between cohorts (log-rank *p* < 0.001). After adjusting for confounders via Cox regression, the vitamin C group demonstrated a 17% reduction in in-hospital mortality risk (adjusted hazard ratio [aHR] 0.67, 95% CI: 0.57–0.79; *p* < 0.001). Consistency was maintained across PSM, paired algorithm, and overlap weighting analyses, with all *p* < 0.001.

**Conclusion:**

Vitamin C supplementation during ICU admission may be associated with reduced in-hospital mortality in SA-AKI patients. These findings underscore the need for prospective randomized trials to validate causality.

## 1 Introduction

Sepsis is clinically defined as a potentially fatal multiorgan dysfunction resulting from a dysregulated host response to infection, which contributes to substantial morbidity and mortality burdens in critically ill populations (1). Global epidemiological data indicate sepsis affects nearly 50 million cases annually, accounting for approximately 11 million fatalities worldwide (2). Renal dysfunction frequently manifests as the earliest and most severe organ involvement in septic patients. Acute kidney injury (AKI), a clinically heterogeneous syndrome characterized by rapid deterioration of glomerular filtration rate (GFR), demonstrates phenotypic variability extending beyond traditional acute renal failure paradigms (3). Importantly, sepsis-associated AKI (SA-AKI) is independently associated with increased hazards for in-hospital mortality, accelerated progression to chronic kidney disease, and persistent renal replacement therapy (RRT) dependency (4, 5).

Emerging evidence has delineated the pleiotropic mechanisms underlying vitamin C’s therapeutic potential in sepsis management, encompassing antioxidant, anti-inflammatory, microvascular stabilizing, and cytoprotective properties (6–9). Functioning as a potent electron donor, this micronutrient effectively neutralizes reactive oxygen species (ROS), thereby reducing lipid peroxidation cascade, preventing DNA strand breaks, and maintaining podocyte cytoskeletal architecture through glutathione regeneration pathways (10, 11). Preclinical studies substantiate its nephroprotective efficacy, particularly in attenuating vancomycin-induced acute tubular necrosis via Nrf2-mediated oxidative stress mitigation (12). Mechanistically, vitamin C exerts immunomodulatory effects through suppression of NF-κB nuclear translocation, subsequent downregulation of proinflammatory mediators (TNF-α, IL-6, HMGB1), induction of M2 macrophage polarization via STAT6 activation, and regulation of neutrophil extracellular trap (NET) formation (11–13). The dual hemodynamic benefits are achieved through enhanced nitric oxide synthase (NOS) coupling for endothelial-dependent vasodilation, concomitantly facilitating catecholamine biosynthesis by serving as an essential cofactor for dopamine β-hydroxylase, which potentiates α1-adrenergic receptor responsiveness to vasoactive agents.

Despite robust preclinical evidence supporting vitamin C’s antioxidant, immunomodulatory, and organ-protective effects in sepsis, its clinical efficacy remains debated. While a review suggests potential mortality reduction in sepsis, concerns have emerged regarding increased risks of death or organ dysfunction in adults receiving vasopressors and intravenous vitamin C therapy (14). Shao et al. (15) reported dose-dependent mortality risks with vitamin C, and meta-analyses reveal conflicting conclusions on combination therapies due to heterogeneous patient populations and variable treatment protocols (16). The C-EASIE trial by Vandervelden et al. (17) found no significant improvement in 28-day mortality or organ function with early vitamin C use. Existing studies predominantly focus on general sepsis populations, overlooking sepsis-associated acute kidney injury (SA-AKI) patients—a high-risk subgroup with distinct pathophysiology. Our study utilizes a real-world ICU database to investigate vitamin C’s association with outcomes in SA-AKI patients through propensity score analyses, aiming to inform targeted interventions and clinical trial design.

## 2 Methods

### 2.1 Data source

This population-based cohort study utilized the Medical Information Mart for Intensive Care IV (MIMIC-IV v3.0), an expanded critical care database containing 76,540 ICU admissions from 2008 to 2019. Data access was authorized (Certification ID: 13278787), with ethical approvals granted by the MIT Institutional Review Board (No. 0403000206) and Beth Israel Deaconess Medical Center (2001-P-001699/14). All data were de-identified prior to analysis (18).

### 2.2 Study population

From 65,366 patients with initial ICU admissions, we included adults (≥18 years) diagnosed with SA-AKI [Sepsis-3 criteria (19)] and ICU stays ≥48 h. AKI was defined per KDIGO 2012 guidelines (20): serum creatinine (sCr) increase ≥0.3 mg/dL (26.5 μmol/L) within 48 h, sCr ≥ 1.5 × baseline within 7 days, or urine output <0.5 mL/kg/h over 6 h. Baseline sCr was derived from pre-ICU records or the first admission measurement if unavailable.

### 2.3 Exposure and covariates

The primary exposure in this study was defined as the administration of vitamin C supplementation via any route (intravenous or oral) during the ICU stay. This inclusive definition was adopted to comprehensively capture all patients who received vitamin C as part of their clinical management. Patients were categorized into two groups based on their exposure status: those who received vitamin C supplementation during their ICU admission (exposed group), and those who did not receive vitamin C supplementation (non-exposed group). Covariates included in the baseline table comprised demographic characteristics (age, sex, race, BMI), comorbidities (including hypertension, diabetes, congestive heart failure, chronic pulmonary disease, liver disease, and malignancy), lifestyle factors (smoking status), and disease severity scores (SOFA, LODS, SAPS II, Charlson Comorbidity Index, and AKI stage) to adjust for baseline differences and chronic disease burden. In addition, clinical interventions (such as invasive mechanical ventilation, continuous renal replacement therapy, vasopressors, immunosuppressants, glucocorticoids, and antihypertensive agents), vital signs (temperature, heart rate, blood pressure, and oxygenation indices), and laboratory parameters (including lactate, blood cell counts, albumin, creatinine, blood urea nitrogen, coagulation indices, liver function tests, glucose, and urine output) were incorporated to account for acute physiological status and organ function.

### 2.4 Outcomes

Primary outcome: In-hospital mortality.

Secondary outcomes: 28-day mortality.

### 2.5 Statistical analysis

Categorical variables were reported as frequencies (%), continuous variables as mean ± SD or median (IQR). Group comparisons employed chi-square, t-, or Kruskal-Wallis tests. Survival analysis used Kaplan–Meier curves with log-rank tests.

To address confounding, 1:1 propensity score matching (PSM, caliper = 0.05) incorporated age, sex, race, vitals, labs, comorbidities, and SOFA scores. Covariate balance was verified via standardized mean differences (SMD < 0.1). Multivariable Cox regression adjusted for propensity score-weighted covariates. Sensitivity analyses included paired algorithm (PA) (21) and overlap weighting (OW) models (22). Subgroup analyses stratified by age, sex, SOFA, interventions, and comorbidities. Analyses utilized R v4.3.3.

## 3 Results

### 3.1 Participant selection

**Figure 1:**
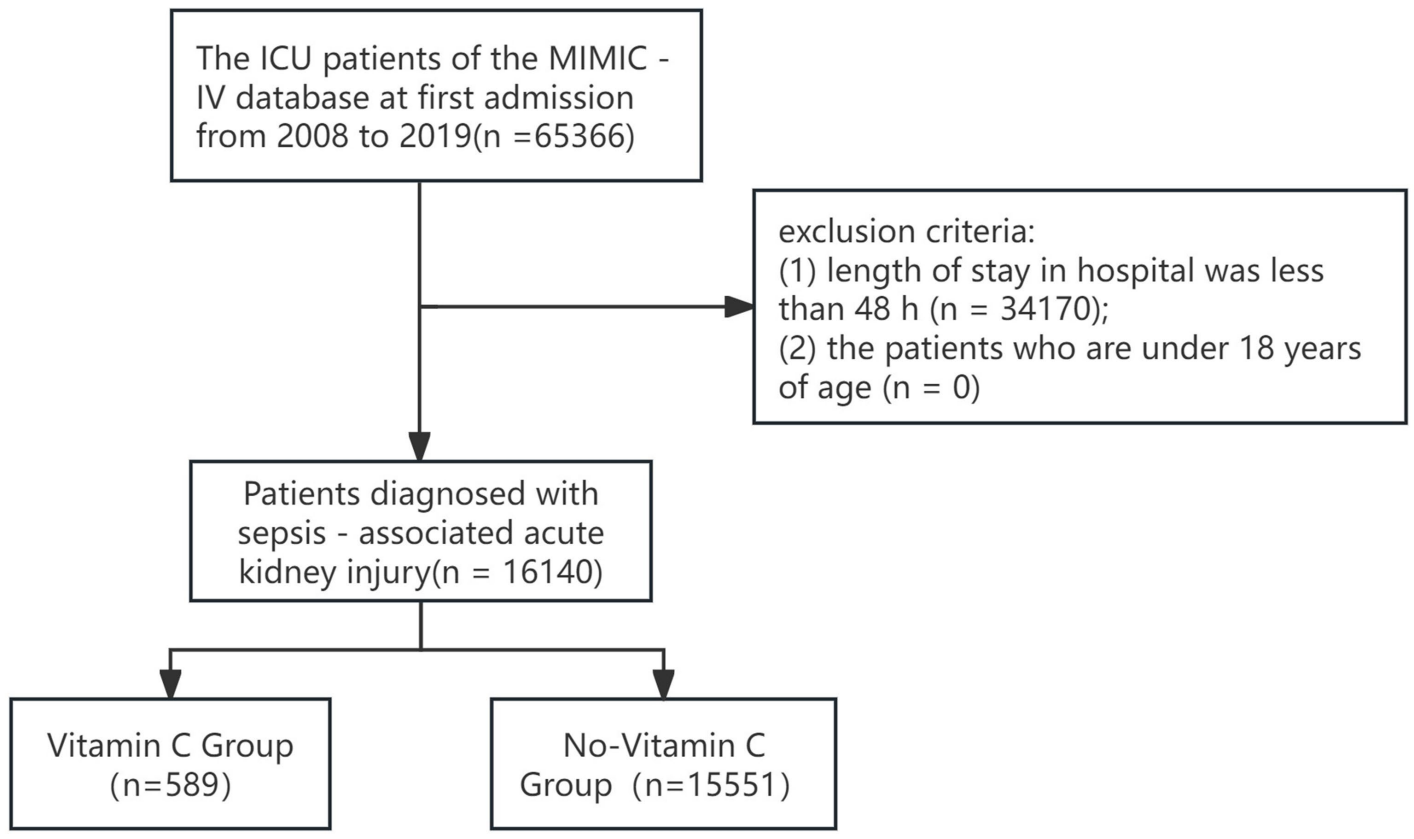
Flowchart of participant selection.

From 65,366 initial ICU admissions, 16,140 SA-AKI patients were enrolled (Figure 1), including 589 vitamin C recipients and 15,551 non-recipients.

### 3.2 Baseline characteristics

Pre-PSM, the vitamin C group exhibited higher BUN levels, comorbidities (congestive heart failure, chronic lung disease, severe liver disease, diabetes), Charlson indices, and intervention requirements (CRRT, mechanical ventilation). Post-PSM, covariates were balanced across groups (Table 1).

**Table 1:** Baseline characteristics of participants.

Covariate	Unmatched patients			*p*-value	Propensity-score-matched patients			*P*-value
	Total	No Vitamin C	Vitamin C		Total	No Vitamin C	Vitamin C	
*n*	16,140	15,551	589		1,072	536	536	
Gender = 1 (%)	9,323 (57.76)	8,978 (57.73)	345 (58.57)	0.7164	617 (57.6)	309 (57.6)	308 (57.5)	1
Race (%)				0.1861				0.293
White	10,267 (63.61)	9,902 (63.67)	365 (61.97)		682 (63.6)	347 (64.7)	335 (62.5)	
Black	1,356 (8.40)	1,292 (8.31)	64 (10.87)		116 (10.8)	60 (11.2)	56 (10.4)	
Asian	404 (2.50)	392 (2.52)	12 (2.04)		23 (2.1)	11 (2.1)	12 (2.2)	
Hispanic	505 (3.13)	483 (3.11)	22 (3.74)		27 (2.5)	8 (1.5)	19 (3.5)	
Others	3,608 (22.35)	3,482 (22.39)	126 (21.39)		224 (20.9)	110 (20.5)	114 (21.3)	
Hospital_death (%)	3,261 (20.20)	3,110 (20.00)	151 (25.64)	0.001	251 (23.4)	122 (22.8)	129 (24.1)	0.665
Smoker (%)	1,095 (6.78)	1,063 (6.84)	32 (5.43)	0.213	62 (5.8)	30 (5.6)	32 (6.0)	0.896
Myocardial_infarct (%)	3,025 (18.74)	2,913 (18.73)	112 (19.02)	0.9051	222 (20.7)	117 (21.8)	105 (19.6)	0.407
Congestive_heart_failure (%)	5,271 (32.66)	5,038 (32.40)	233 (39.56)	0.0003	435 (40.6)	221 (41.2)	214 (39.9)	0.709
Peripheral_vascular disease (%)	2081 (12.89)	2007 (12.91)	74 (12.56)	0.8566	140 (13.1)	67 (12.5)	73 (13.6)	0.65
Cerebrovascular_disease (%)	2,914 (18.05)	2,813 (18.09)	101 (17.15)	0.5973	189 (17.6)	101 (18.8)	88 (16.4)	0.336
Dementia (%)	702 (4.35)	676 (4.35)	26 (4.41)	1	58 (5.4)	32 (6.0)	26 (4.9)	0.5
Chronic_pulmonary_disease (%)	4,327 (26.81)	4,166 (26.79)	161 (27.33)	0.8058	312 (29.1)	159 (29.7)	153 (28.5)	0.737
Mild liver disease (%)	2,586 (16.02)	2,502 (16.09)	84 (14.26)	0.2586	159 (14.8)	80 (14.9)	79 (14.7)	1
Diabetes (%)	5,092 (31.55)	4,871 (31.32)	221 (37.52)	0.0017	394 (36.8)	196 (36.6)	198 (36.9)	0.949
Malignant cancer (%)	2,149 (13.31)	2085 (13.41)	64 (10.87)	0.0854	132 (12.3)	71 (13.2)	61 (11.4)	0.403
Severe liver disease (%)	1,368 (8.48)	1,312 (8.44)	56 (9.51)	0.4006	94 (8.8)	44 (8.2)	50 (9.3)	0.589
Aids (%)	92 (0.57)	91 (0.59)	1 (0.17)	0.3004	3 (0.3)	2 (0.4)	1 (0.2)	1
Hypertension (%)	6,584 (40.79)	6,405 (41.19)	179 (30.39)	<0.0001	344 (32.1)	175 (32.6)	169 (31.5)	0.744
InvasiveVent (%)	11,553 (71.58)	11,110 (71.44)	443 (75.21)	0.0518	778 (72.6)	387 (72.2)	391 (72.9)	0.837
Crrt (%)	1,633 (10.12)	1,471 (9.46)	162 (27.50)	<0.0001	230 (21.5)	114 (21.3)	116 (21.6)	0.941
Immunosuppressant (%)	645 (4.00)	617 (3.97)	28 (4.75)	0.3958	43 (4.0)	21 (3.9)	22 (4.1)	1
Glucocorticoid (%)	4,031 (24.98)	3,808 (24.49)	223 (37.86)	<0.0001	370 (34.5)	183 (34.1)	187 (34.9)	0.847
Antihypertensive (%)	12,929 (80.11)	12,426 (79.90)	503 (85.40)	0.0013	902 (84.1)	448 (83.6)	454 (84.7)	0.676
Norepinephrine (%)	4,717 (29.23)	4,399 (28.29)	318 (53.99)	<0.0001	546 (50.9)	281 (52.4)	265 (49.4)	0.359
Age (median [IQR])	68.188 [56.882, 78.956]	68.156 [56.843, 78.986]	68.812 [58.290, 78.280]	0.7369	69.12 [58.59, 78.80]	68.79 [58.54, 78.84]	69.30 [58.60, 78.74]	0.891
SOFA (median [IQR])	7.000 [5.000, 10.000]	7.000 [5.000, 10.000]	9.000 [6.000, 12.000]	<0.0001	8.50 [6.00, 12.00]	8.50 [6.00, 12.00]	8.50 [6.00, 12.00]	0.684
Lods (median [IQR])	6.000 [4.000, 8.000]	6.000 [4.000, 8.000]	7.000 [5.000, 8.000]	<0.0001	6.00 [4.00, 8.00]	6.00 [4.00, 9.00]	6.00 [4.00, 8.00]	0.502
Charlson (median [IQR])	5.000 [3.000, 7.000]	5.000 [3.000, 7.000]	5.000 [4.000, 8.000]	0.0932	5.00 [4.00, 8.00]	5.00 [4.00, 7.00]	5.00 [4.00, 8.00]	0.933
Sapsii (median [IQR])	41.000 [32.000, 51.000]	40.000 [32.000, 51.000]	43.000 [34.000, 53.000]	0.0011	42.00 [34.00, 53.00]	42.00 [34.00, 53.00]	42.00 [34.00, 52.00]	0.992
Temperature (median [IQR])	36.720 [36.390, 37.110]	36.720 [36.390, 37.110]	36.780 [36.390, 37.170]	0.1652	36.78 [36.39, 37.11]	36.78 [36.44, 37.11]	36.72 [36.39, 37.11]	0.321
Heart_rate (median [IQR])	89.000 [77.000, 104.000]	89.000 [77.000, 104.000]	90.000 [78.000, 105.000]	0.2443	90.00 [78.00, 106.00]	90.50 [78.75, 107.00]	89.00 [78.00, 105.25]	0.474
SBP (median [IQR])	120.000 [104.000, 138.000]	120.000 [104.750, 139.000]	117.000 [102.000, 134.000]	0.0005	118.00 [102.00, 136.00]	119.00 [102.00, 136.00]	118.00 [102.00, 134.25]	0.458
DBP (median [IQR])	66.000 [55.000, 78.000]	66.000 [55.000, 78.000]	63.000 [53.000, 75.000]	0.0006	64.00 [53.00, 76.00]	65.00 [54.00, 78.00]	63.00 [53.00, 75.00]	0.152
MBP (median [IQR])	81.000 [70.000, 94.000]	81.000 [70.000, 94.000]	78.000 [69.000, 90.000]	0.001	79.00 [69.00, 92.00]	80.00 [69.00, 93.00]	78.00 [69.00, 90.00]	0.222
Pao_2_/Fio_2_ (median [IQR])	240.000 [150.000, 348.000]	240.000 [151.000, 348.333]	223.333 [126.667, 340.000]	0.0086	229.17 [136.19, 338.08]	226.75 [141.92, 332.50]	230.50 [129.14, 343.50]	0.962
SO_2_(median [IQR])	97.000 [95.000, 98.000]	97.000 [95.000, 98.000]	97.000 [95.000, 98.000]	0.0228	97.00 [95.00, 98.00]	97.00 [94.75, 98.00]	97.00 [95.00, 98.00]	0.729
Lactate (median [IQR])	1.600 [1.100, 2.500]	1.600 [1.100, 2.500]	1.600 [1.100, 2.600]	0.4185	1.60 [1.10, 2.60]	1.50 [1.10, 2.60]	1.60 [1.10, 2.62]	0.599
WBC (median [IQR])	11.800 [8.300, 16.500]	11.800 [8.300, 16.500]	11.600 [7.800, 16.400]	0.2182	11.80 [8.10, 16.00]	12.10 [8.38, 16.00]	11.60 [7.77, 16.10]	0.298
Neutrophils_abs (median [IQR])	9.860 [6.520, 14.291]	9.870 [6.530, 14.293]	9.547 [6.207, 14.190]	0.4147	9.93 [6.53, 14.38]	10.49 [6.76, 14.29]	9.53 [6.20, 14.43]	0.254
Monocytes (median [IQR])	4.600 [3.000, 7.000]	4.600 [3.000, 7.000]	5.000 [3.000, 7.500]	0.012	5.00 [3.10, 7.40]	5.00 [3.18, 7.40]	5.00 [3.00, 7.50]	0.854
Platelet (median [IQR])	186.000 [129.000, 256.000]	186.000 [129.000, 256.000]	200.000 [131.000, 281.000]	0.0163	200.00 [128.75, 268.00]	200.50 [128.00, 263.25]	196.50 [129.00, 274.50]	0.821
Albumin (median [IQR])	3.000 [2.600, 3.500]	3.000 [2.600, 3.500]	3.000 [2.500, 3.400]	0.0015	3.00 [2.50, 3.40]	2.90 [2.50, 3.40]	3.00 [2.50, 3.40]	0.96
Creatinine (median [IQR])	1.100 [0.800, 1.700]	1.100 [0.800, 1.700]	1.200 [0.800, 2.100]	<0.0001	1.20 [0.80, 2.00]	1.20 [0.80, 2.00]	1.20 [0.80, 2.00]	0.942
BUN (median [IQR])	22.000 [14.000, 36.000]	22.000 [14.000, 36.000]	26.000 [17.000, 47.000]	<0.0001	25.00 [16.00, 44.00]	24.00 [16.00, 44.25]	26.00 [16.00, 44.00]	0.536
Calcium total (median [IQR])	8.300 [7.700, 8.800]	8.300 [7.700, 8.800]	8.300 [7.800, 8.800]	0.8552	8.20 [7.70, 8.80]	8.20 [7.70, 8.80]	8.30 [7.80, 8.80]	0.587
PT (median [IQR])	14.500 [12.800, 17.400]	14.500 [12.700, 17.400]	14.800 [13.100, 18.000]	0.0021	14.80 [13.00, 18.00]	14.70 [12.80, 18.00]	14.85 [13.10, 18.00]	0.348
PTT (median [IQR])	31.300 [27.300, 39.400]	31.300 [27.300, 39.300]	32.100 [28.300, 40.600]	0.0253	31.95 [27.80, 40.15]	31.75 [27.50, 40.02]	32.10 [28.40, 40.35]	0.42
Inr (median [IQR])	1.300 [1.200, 1.600]	1.300 [1.200, 1.600]	1.300 [1.200, 1.600]	0.0012	1.30 [1.20, 1.60]	1.30 [1.20, 1.60]	1.30 [1.20, 1.60]	0.327
Fibrinogen (median [IQR])	275.000 [191.000, 434.000]	273.000 [190.000, 430.500]	325.000 [215.000, 502.000]	<0.0001	320.00 [213.75, 497.50]	324.00 [217.75, 508.00]	315.50 [209.00, 480.50]	0.153
Alt (median [IQR])	29.000 [17.000, 67.000]	30.000 [17.000, 67.000]	27.000 [17.000, 56.000]	0.0556	27.00 [17.00, 57.00]	28.50 [17.00, 58.00]	27.00 [16.00, 54.00]	0.478
Ast (median [IQR])	45.000 [25.000, 101.000]	45.000 [25.000, 102.000]	44.000 [26.000, 87.000]	0.3697	44.00 [25.00, 86.25]	45.00 [25.00, 86.00]	43.00 [25.00, 87.25]	0.667
Bilirubin_total (median [IQR])	0.700 [0.400, 1.400]	0.700 [0.400, 1.400]	0.700 [0.400, 1.400]	0.5773	0.70 [0.40, 1.40]	0.70 [0.40, 1.40]	0.70 [0.40, 1.40]	0.863
Glucose (median [IQR])	130.000 [105.000, 171.000]	130.000 [105.000, 171.000]	131.000 [106.000, 168.000]	0.9255	132.00 [107.00, 170.00]	132.00 [108.75, 171.25]	131.00 [104.00, 169.00]	0.385
Urine output (median [IQR])	326.000 [150.000, 600.000]	330.000 [150.000, 600.000]	285.000 [115.000, 560.000]	0.0004	280.00 [120.00, 550.00]	270.00 [125.00, 521.25]	297.50 [120.00, 580.00]	0.665
BMI (median [IQR])	27.888 [24.088, 33.025]	27.845 [24.074, 32.976]	28.466 [24.587, 34.937]	0.0087	28.47 [24.42, 34.67]	28.82 [24.54, 34.72]	28.06 [24.25, 34.44]	0.144
Aki stage (%)				<0.0001				0.813
1	2,833 (17.55)	2,764 (17.77)	69 (11.71)		142 (13.2)	73 (13.6)	69 (12.9)	
2	7,461 (46.23)	7,231 (46.50)	230 (39.05)		440 (41.0)	215 (40.1)	225 (42.0)	
3	5,846 (36.22)	5,556 (35.73)	290 (49.24)		490 (45.7)	248 (46.3)	242 (45.1)	

AKI, acute kidney injury; CRRT, continuous renal replacement therapy; SOFA, sequential organ failure assessment; SAPS II, simplified acute physiology score II; MBP, mean arterial pressure; PaO₂/FiO₂, partial pressure of arterial oxygen to fraction of inspired oxygen ratio; SpO₂, peripheral oxygen saturation; WBC, white blood cell count; BUN, blood urea nitrogen; PT, prothrombin time; PTT, partial thromboplastin time; INR, international normalized ratio; ALT, alanine aminotransferase; AST, aspartate aminotransferase.

### 3.3 Primary outcomes

**Figure 2:**
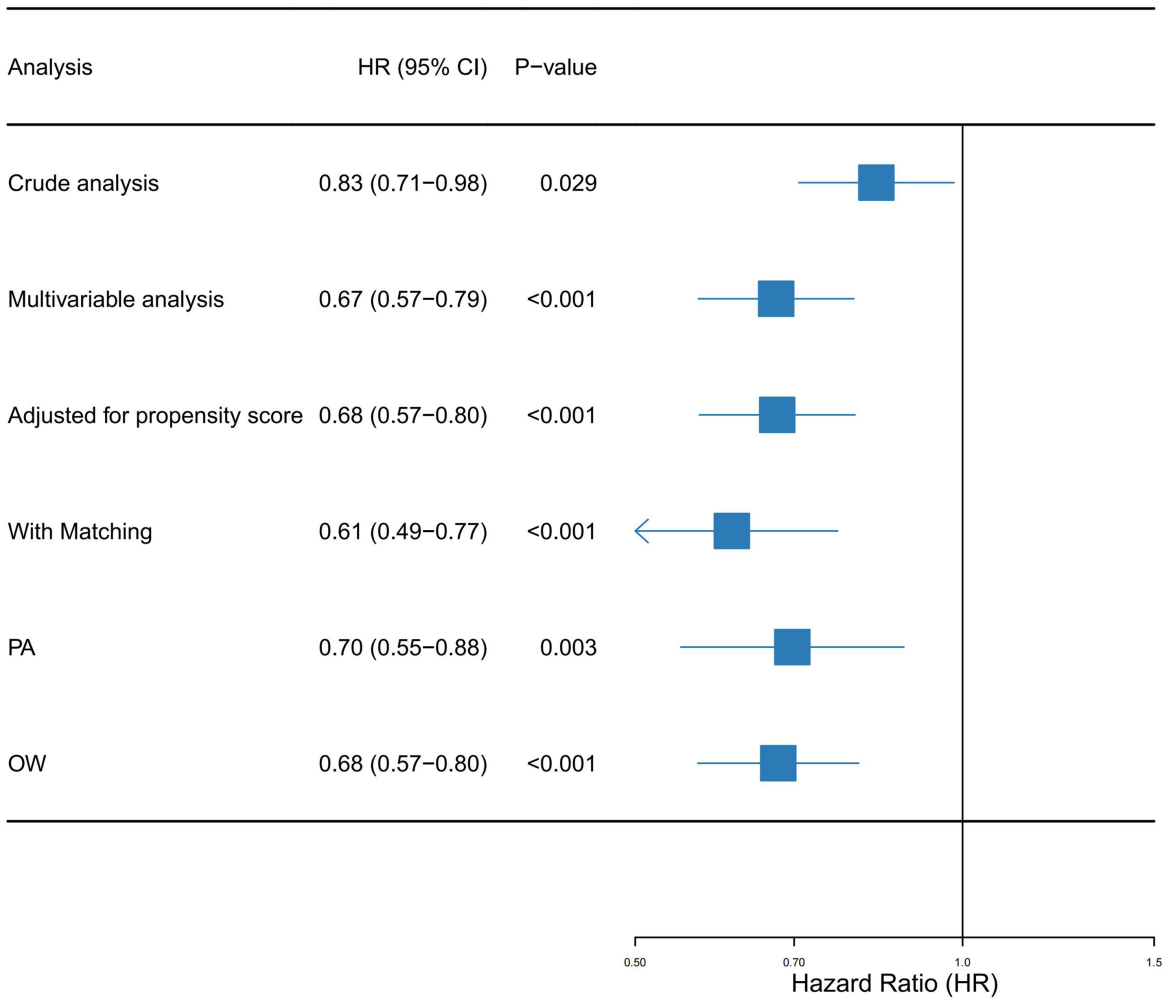
Kaplan–Meier survival curve for in-hospital mortality according to different groups.

The overall in-hospital mortality rate was 18.8%. Subgroup analysis revealed distinct mortality patterns: the vitamin C supplementation group exhibited significantly lower in-hospital mortality (20.0%, 151/589) compared to the non-supplementation group (25.6%, 3,110/15,551). Kaplan–Meier survival curves demonstrated a pronounced divergence in 28-day mortality favoring the vitamin C cohort (Figure 2).

Univariate Cox regression identified vitamin C supplementation as a protective factor against mortality in SA-AKI patients, yielding a hazard ratio (HR) of 0.83 (95% CI: 0.71–0.98). This corresponds to an estimated 17% reduction in mortality risk among supplementation recipients. Multivariable Cox regression incorporating all covariates from Table 1 strengthened this association (adjusted HR: 0.67, 95% CI: 0.57–0.79). Propensity score-matched analyses further validated these findings: PSM-adjusted HR: 0.68 (95% CI: 0.57–0.80); Overlap weighting model HR: 0.61 (95% CI: 0.49–0.77).

**Figure 3:**
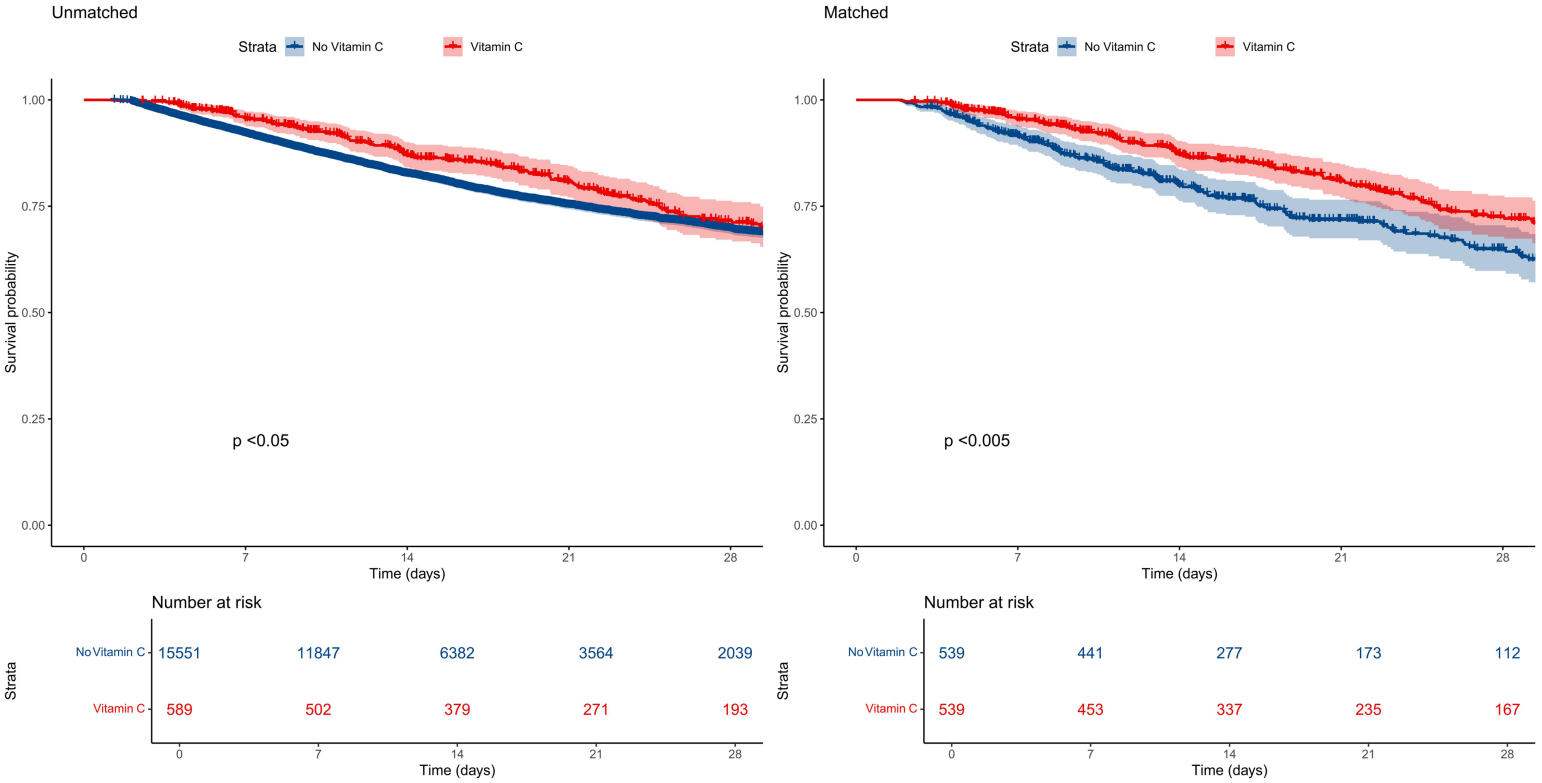
Forest plot shows HRs of in-hospital mortality in vitamin D group using a variety of models.

Sensitivity analyses using paired algorithm (PA) and overlap weighting (OW) methodologies consistently reinforced the stability of this protective effect (Figure 3). The concordance across multiple statistical approaches underscores the reliability of the observed mortality reduction.

### 3.4 Subgroup analysis

**Figure 4:**
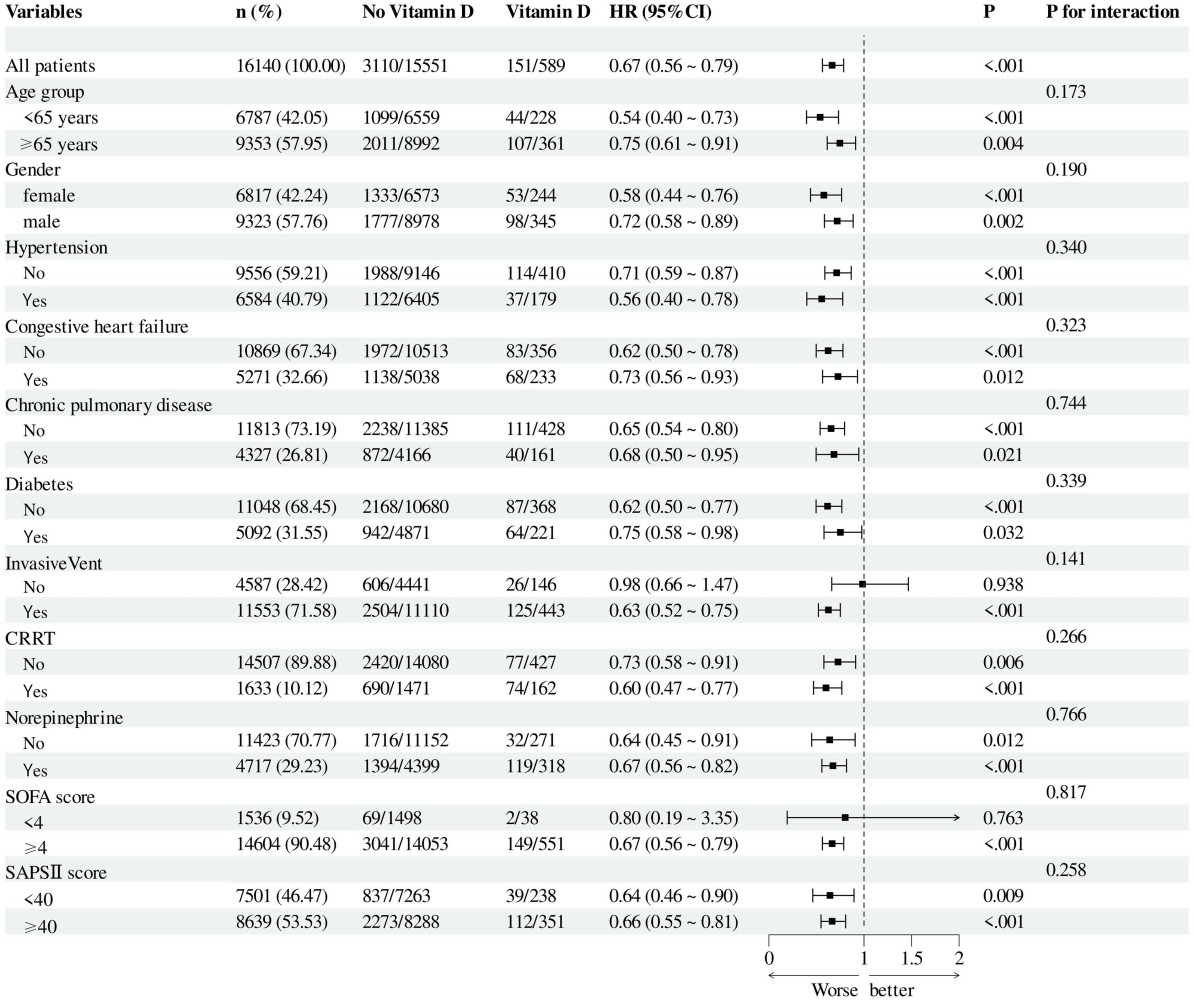
Forest plot shows HRs of in-hospital mortality in vitamin D group in subgroup analyses.

After adjusting for all covariates in Table 1, subgroup analyses were performed based on age, sex, Sequential Organ Failure Assessment (SOFA) score, Simplified Acute Physiology Score II (SAPS II), vasopressor use, presence of invasive mechanical ventilation, and comorbidities including hypertension, diabetes mellitus, chronic kidney disease, chronic pulmonary disease, and congestive heart failure. The results remained consistent across all subgroups (Figure 4).

## 4 Discussion

Our study demonstrates that in-hospital vitamin C supplementation is associated with a reduced short-term mortality risk in SA-AKI patients, consistent with previous observational studies. The multifaceted renoprotective mechanisms may involve several canonical and recently elucidated pathways. Recent single-cell transcriptomic studies have revealed that vitamin C can restore electron transport chain complex assembly in proximal tubular cells through the upregulation of mitochondrial chaperones (HSP60/70) and prohibitin-1 (PHB1), which are crucial for cristae morphogenesis during sepsis-induced bioenergetic crisis (23). In addition, vitamin C enhances TET2-mediated 5hmC modification at GPX4 promoter regions, counteracting sepsis-induced DNA hypermethylation (mean methylation level decreased by 37.2%, *p* = 0.009), thereby preserving glutathione peroxidase 4 (GPX4) activity and significantly reducing renal lipid peroxidation markers such as 4-HNE by 54% compared to controls (23). Dynamic contrast-enhanced ultrasonography provides further evidence that vitamin C treatment increases the renal cortical microvascular flow index (MFI) by 1.8-fold (*p* < 0.01), an effect mechanistically linked to the suppression of PAD4-dependent histone citrullination (62% reduction) and subsequent limitation of neutrophil extracellular trap (NET) formation, thus improving microvascular perfusion heterogeneity (24). Moreover, recent animal model data indicate that vitamin C reverses sepsis-induced gut dysbiosis, increasing the abundance of short-chain fatty acid–producing bacteria such as Roseburia spp. by 3.2-fold, which, through GPR43 receptor activation, enhances tubular autophagic flux (LC3-II/I ratio increased by 2.1-fold) (25, 26).

These novel insights complement established mechanisms whereby vitamin C activates the Nrf2/ARE pathway, enhancing the expression of phase II detoxifying enzymes (HO-1, NQO1), particularly in proximal tubular epithelial cells where oxidative damage is most severe (27). Vitamin C also promotes mitochondrial biogenesis through PGC-1α upregulation, restores ATP production during ischemic injury (28), and inhibits ferroptosis via preservation of GPX4 activity, effectively reducing lipid peroxidation markers like 4-HNE in renal tissue (29). Notably, the immunomodulatory effects of vitamin C exhibit temporal specificity—early enhancement of M1 macrophage bactericidal capacity (evidenced by IL-12 elevation within 24 h) is followed by M2 polarization promoting tissue repair (increased IL-10 at 72 h) (30). This biphasic regulation may explain the reduced vasopressor duration observed in our cohort. Furthermore, vitamin C’s ability to suppress NETosis through PAD4 inhibition could mitigate microvascular thrombosis, a critical pathomechanism in SA-AKI (31).

Vitamin C, which exists in multiple biological forms and is primarily obtained through dietary intake, undergoes hepatic metabolism as its central regulatory pathway. Its deficiency has been mechanistically linked to heightened infection susceptibility, particularly during bacterial and viral challenges (32). Beyond its canonical antioxidant properties, vitamin C exerts pleiotropic effects through enzymatic cofactor roles in tissue repair (33) and endothelial function modulation via oxidative stress mitigation (34). Clinical evidence suggests that intravenous vitamin C administration in critical infections (e.g., sepsis) reduces organ dysfunction and improves survival (35), while enhancing immune cell-mediated pathogen clearance (36). Paradoxically, vitamin C deficiency may exacerbate cardiovascular risks through dyslipidemia and vascular dysregulation (37), underscoring its systemic importance in critical illness (38).

Vitamin C exerts critical immunoregulatory effects in sepsis pathophysiology, mediating the balance between initial pathogen clearance and subsequent hyperinflammation-induced organ failure (30). Experimental models confirm its dual capacity to attenuate cytokine storms while maintaining antimicrobial defenses through three mechanisms: enhanced neutrophil phagocytosis, endothelial barrier stabilization, and NLRP3 inflammasome suppression (39–41). Clinical meta-analyses reveal geographical heterogeneity in mortality outcomes (42), with developing regions showing 28% risk reduction (43) contrasting with neutral effects in multicenter RCTs (44). Clinical evidence from septic shock patients reveals profound vitamin C depletion with preferential accumulation in immune cells (monocytes: 80 × plasma concentration; granulocytes: 25×). Intravenous supplementation achieves rapid plasma concentration elevation followed by swift decline, indicating active cellular uptake (45). A randomized controlled trial demonstrate combination therapy (vitamin C + hydrocortisone + thiamine) significantly reduces SOFA scores, shortens vasopressor dependence duration, and improves 28-day survival rates, particularly in developing regions (46). Notably, early high-dose regimens (3–4 days) show survival benefits in predefined subgroups (47).

Sepsis-associated AKI (SA-AKI), a prevalent complication with mortality exceeding 40% (48), arises from synergistic hemodynamic, inflammatory, and immunometabolic insults (49). Our findings align with emerging paradigms of SA-AKI as a distinct entity involving mitochondrial dysfunction and immunothrombosis (50). Vitamin C deficiency may exacerbate SA-AKI through impaired redox homeostasis and neutrophil extracellular trap dysregulation (51), compounded by micronutrient interactions in malnourished critically ill patients (52). Early intervention bundles—including antimicrobial stewardship, goal-directed resuscitation, and micronutrient repletion—remain cornerstone strategies (53).

### 4.1 Limitations and future directions

Our study has several limitations requiring cautious interpretation, The MIMIC-IV database lacks detailed records on vitamin C administration patterns (bolus vs. continuous infusion) and concomitant antioxidants use, preventing analysis of dose–response relationships. Previous pharmacokinetic studies suggest plasma concentrations >100 μmol/L require >3 g/d continuous infusion (54), which our data cannot verify. Particularly, the predominance of Caucasian participants (63.6%) raises concerns about generalizability, given ethnic differences in CYP450-mediated vitamin C metabolism (55). Future RCTs should stratify by AKI stage and infection type, while incorporating biomarkers like urinary 8-OHdG to quantify oxidative stress modulation.

## 5 Conclusion

This large-scale retrospective cohort study establishes vitamin C supplementation as an independent predictor of reduced 28-day mortality in ICU-admitted SA-AKI patients, with robust validation through advanced causal inference methods. The intervention’s cost-effectiveness and safety profile support consideration of protocolized vitamin C status monitoring and supplementation in critical care settings. However, therapeutic optimization requires prospective evaluation of dose–response relationships, administration timing, and combination therapies. Large-scale randomized controlled trials are urgently needed to confirm these observational findings and establish evidence-based clinical guidelines.

## Data Availability

The datasets presented in this study can be found in online repositories. The names of the repository/repositories and accession number(s) can be found in the article/supplementary material.
